# Silymarin and Derivatives: From Biosynthesis to Health Benefits

**DOI:** 10.3390/molecules25102415

**Published:** 2020-05-22

**Authors:** Dominique Delmas

**Affiliations:** 1 Faculty of Health Sciences, University of Bourgogne, 7 bd Jeanne d’Arc, F-21000 Dijon, France; dominique.delmas@u-bourgogne.fr; Tel.: +33-380-39-32-26; 2 INSERM Research Center U1231–Cancer and Adaptive Immune Response Team, Bioactive Molecules and Health Research Group, F-21000 Dijon, France; 3 Centre anticancéreux Georges François Leclerc Center, F-21000 Dijon, France

**Keywords:** silymarin, silybin, bioavailability, formulation, antioxidant, cancer, inflammation, age-related degenerative diseases

The past decade has been marked by an intense scientific interest in the use of compounds or micronutrients of natural origin and their potential effects on human health, both from researchers and industry. These phytomolecules have cellular targets similar to those of the new drugs developed by pharmaceutical companies. Indeed, more than 1600 patents are currently reported relating to flavonoids and 3000 patents relating to polyphenols. Pleiotropic pharmaceutical activities are claimed in fields such as cancer, inflammation, arthritis, cardiovascular diseases, auto-immune diseases, eye diseases, and many other domains. Among micronutrients and plant-derived compounds, flavonolignans are a family of natural products present in plants, composed of a flavonoid moiety and a phenylpropanoid or lignan part, that could contribute to new strategies to fight various modern pathologies, and thus participate in preventive strategies. In the mid-1970s, Michael Sporn was the first to define the term “chemoprevention” as a strategy using natural or chemical substances to inhibit, reverse or delay the multistage process of carcinogenesis with relative nontoxicity to normal cells. Since then, this concept has evolved considerably and is no longer limited solely to the prevention of cancer, as initially described by Sporn, but extends to the prevention of the occurrence of many frequent pathologies such as diabetes, atherosclerosis, diseases with an inflammatory component, whether chronic or acute, and even degenerative diseases. Among natural bioactive molecules, and in particular flavonoids, silymarin, the extract of milk thistle, *Silybum marianum* (L.) Gaertn. (*Asteraceae*), and its major active flavonolignan silybin (or silibinin), could constitute a candidate of choice to exert a preventive action against many evils of our century. Indeed, silymarin has been used for more than 2000 years as a functional food ingredient for the treatment of a large number of liver disorders. It is now used in Europe as complementary protection in patients receiving medication known to cause liver problems. The past five years have been marked by a revival of publications concerning silymarin, with more than 2670 citations in 2019, representing an almost 2556 increase in citations in four years (Figure 1A). A wide range of therapeutic properties have been proposed in the 1208 records for silymarin in the Web of Science (Figure 1B), including antioxidant, anti-inflammatory, anti-cancer, and anti-viral activities, as well as its potential usefulness in the treatment of several liver disorders, such as chronic liver diseases, cirrhosis, and hepatocellular carcinoma.

This Special Issue is dedicated to the current knowledge of biosynthesis and health properties of silymarin and its derivatives, as well as the latest scientific advances in various domains, including bioavailability, cancer, inflammation and immunity, age-related degeneration and metabolism-related pathologies.

First of all, Shah and colleagues report a very interesting article showing the involvement of light and melatonin on the biosynthesis of silymarin where they demonstrate that a continuous light associated with melatonin increased total flavonoid and total phenolic content with an increase of the level of silybins (A and B), silydianin, isolsilychristin, and silychristin [1]. Like numerous natural molecules, silymarin has very low bioavailability, as shown for many polyphenols, which could limit its biological action. In their review, Xie and colleagues highlight that some compounds (i.e., tangeretin, piperine, and baicalein), known to inhibit the efflux transporters of multidrug resistance-associated protein (MRP2) and breast cancer resistance protein (BCRP), can enhance the absorption and activity of silybin [2]. Furthermore, in the context of improving silymarin bioavailability and thus its biological activities, other approaches are proposed, in particular through new formulations such as the production of nanomicelles to improve the solubility and oral absorption of silymarin. For example, in their article, Piazzini et al. describe the use of nanotechnological strategies (particularly nanocrystals, nanosupensions or complexes, with cyclodextrins and phospholipids) to potentiate the therapeutic action and promote the sustained release of silymarin [3]. Di Costanzo and Angelico review the different nanostructured systems available in the literature as delivery strategies to improve the absorption and bioavailability of silymarin [4]. Similarly, for a dermal formulation, Esposito et al. describe in their original article, a new water-soluble microencapsulated milk thistle extract [5]. Indeed, silymarin could protect against Ultraviolet A (UVA) exposure due to its ability to scavenge reactive oxygen species (ROS), as shown by Vostalova and coworkers [6]. Moreover, in silico predictions can be usefully applied to estimate general lipophilicity trends, such as skin penetration or accumulation predictions, as demonstrated by Kosina and colleagues [7]. Nevertheless, Fidrus et al. warn of the use of silymarin in the context of dermoprotection, since this flavonoid may be capable of inducing photolesions and adverse impacts [8]. In this way, Juranova et al. show that 2,3-dehydrosilybin could both diminish interleukin-6 and interleukin-8 secretion induced by exposure of lipopolysaccharide on human dermal fibroblasts but could upregulate interleukin-8 mRNA associated with NF-κB and AP-1 activation [9]. Other reports have developed a new class of synthetic flavonolignans such as the 3-*O*-palmitoyl-silybin, where Drouet and colleagues show an important anti-lipoperoxidant activity [10]. These recent developments in the synthesis, characterization, and antioxidants activity of a new class of synthetic flavonolignans are discussed by Romanucci et al. in their review [11].

Despite its low bioavailability, scientific evidence continues to highlight the biological relevance of silymarin to human health, mainly in numerous pathologies where oxidative stress and inflammation play a preponderant role. Indeed, in the context of non-alcoholic steatohepatitis (NASH), silymarin is able to reduce ROS generation, as shown by Anfuso and coworkers [12]. In another context, namely the nervous system, Guo et al. summarize in particular how silymarin can control the production of Aβ by inhibiting the precursor substance of Aβ (β-amyloid precursor protein), and how it is able to inhibit the polymerization of Aβ, thereby providing good prospects for the treatment of Alzheimer’s disease [13]. With regard to inflammation, which plays a crucial role through the action of various pro-inflammatory cytokines or the activation of inflammatory complexes such as the NLRP1 and NLRP3 complexes, Matias et al. demonstrate in their studies that silibinin can modulate the sterile inflammation established in monocytes from preeclamptic women via downregulation of the NFκB pathway and activation of NLRP1/NLRP3 inflammasomes [14].

In addition to these antioxidant and anti-inflammatory properties, silymarin also has interesting properties such as anti-viral and anti-cancer activities. In this regard, Liu et al. report the anti-viral effect of silymarin-associated drugs in chronic hepatitis C, liver transplantation, and difficult-to-treat HIV/HCV-coinfected patients [15]. On the other side, Delmas and their colleagues summarize in their review the current knowledge on the potential targets of silymarin against various cancers and the ability of this flavonolignan to act as a chemopreventive agent [16]. Furthermore, the synthesis of silymarin derivatives seems very promising to inhibit cancer cell proliferation, as demonstrated by Vue et al. in prostate cancer cells [17]. Finally, silymarin may also be useful as a therapeutic adjuvant, since it is frequently prescribed with other medications such as methotrexate, which has significant liver toxicity, and could be used as a chemosensitizing agent against cancer progression [16].

In view of the recent studies and reviews published in this Special Issue, there is no doubt that silymarin and its derivatives have many positive effects that may be of significant relevance, both in the field of prevention and in the search for new therapeutic strategies. Future clinical studies will confirm the enthusiasm for this natural flavonolignan.

## Figures and Tables

**Figure 1:**
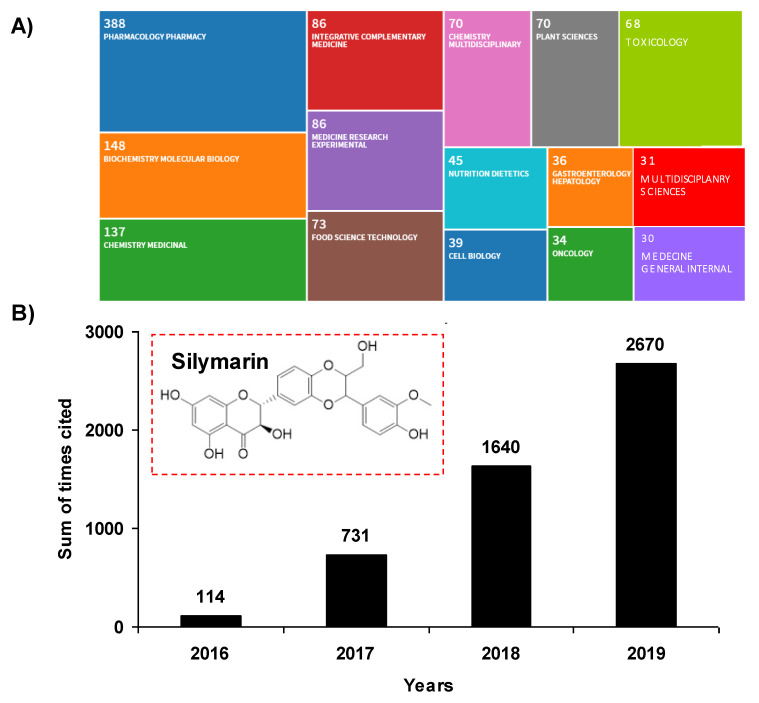
(**A**) **Tree map of fields for silymarin terms.** The record count in each rectangle is the total number of articles published in the last four years (2016 to 2020). The count includes Early Access articles that are fully peer-reviewed, citable, and published. (**B**) **Sum of times cited.** This is the total number of citations to silymarin in the results set. **Inset:** chemical structure of silymarin.
